# Benefit sharing: Why inclusive provenance metadata matter

**DOI:** 10.3389/fgene.2022.1014044

**Published:** 2022-09-21

**Authors:** Jacob Golan, KatieLee Riddle, Maui Hudson, Jane Anderson, Natalie Kusabs, Tim Coltman

**Affiliations:** ^1^ School of Law, New York University, New York, NY, United States; ^2^ Te Kotahi Research Institute, University of Waikato, Hamilton, New Zealand; ^3^ Te Kotahi Research Institute, University of Waikato, Hamilton, New Zealand; ^4^ Engelberg Center of Innovation Law and Policy, School of Law, New York University, New York, NY, United States; ^5^ Te Kotahi Research Institute, University of Waikato, Hamilton, New Zealand; ^6^ Waikato Management School, University of Waikato, Hamilton, New Zealand

**Keywords:** provenance, metadata, DSI, benefit sharing, traditional knowledge, Indigenous, TK labels, BC labels

## Abstract

Fair and equitable benefit sharing of genetic resources is an expectation of the Nagoya Protocol. Although the Nagoya Protocol does not yet formally apply to Digital Sequence Information (“DSI”), discussions are currently underway regarding to include such data through ongoing Convention on Biological Diversity (“CBD”) negotiations. While Indigenous Peoples and Local Communities (“IPLC”) expect the value generated from genomic data to be subject to benefit sharing arrangements, a range of views are currently being expressed by Nation States, IPLC and other stakeholders. The use of DSI gives rise to unique considerations, creating a gray area as to how it should be considered under the Nagoya Protocol’s Access and Benefit Sharing (“ABS”) principles. One way for benefit sharing to be enhanced is through the connection of data to proper provenance information. A significant development is the use of digital labeling systems to ensure that the origin of samples is appropriately disclosed. The Traditional Knowledge and Biocultural Labels initiative offers a practical option for data provided to genomic databases. In particular, the BioCultural Labels (“BC Labels”) are a mechanism for Indigenous communities to identify and maintain provenance, origin and authority over biocultural material and data generated from Indigenous land and waters held in research, cultural institutions and data repositories. This form of cultural metadata adds value to the research endeavor and the creation of Indigenous fields within databases adds transparency and accountability to the research environment.

## Background

1.1 Indigenous lands, resources and Traditional Knowledge (“TK”) are regularly the source or subject of academic research. However, Indigenous provenance is most often absent or missing from metadata. Big data in the life sciences can now be generated with relative ease which has exacerbated opportunities for the misappropriation of TK (Curci, 2009). As a result of sidelining Indigenous provenance, unethical misappropriation of material is more likely to occur. This in turn impedes opportunities for the fair and equitable sharing of benefits flowing from TK. Consequently, establishing appropriate attribution and recognition of Indigenous provenance in metadata has emerged as a key mechanism for protecting the rights and interests of Indigenous Peoples and Local Communities (“IPLC”) in relation to their genetic resources (Anderson and Christen, 2019; Anderson and Hudson, 2020; Ambler et al., 2021).

1.2 While IPLC most commonly expect the value generated from biological data to be subject to access and benefit sharing (“ABS”), views expressed by national governments and other stakeholders vary (United Nations Convention on Biological Diversity Ad Hoc Technical Expert Group on Digital Sequence Information on Genetic Resources, 2020; United Nations Convention on Biological Diversity Contact group 5, 2021). Fair and equitable ABS for genetic resources is an expectation of the Nagoya Protocol. Yet, attribution of data from IPLC land and waters has lagged behind global calls to action and despite the clear expectations that data generated within IPLC sovereign territories would be subject to fair and equitable ABS (Golan et al., 2019; United Nations Convention on Biological Diversity Ad Hoc Technical Expert Group on Digital Sequence Information on Genetic Resources, 2020; United Nations Convention on Biological Diversity Contact group 5, 2021).

## The Nagoya Protocol and Indigenous data sovereignty

2.1 In 1992 when the Convention on Biological Diversity (“CBD”) was established, it included the requirement for defining mutually agreed terms (“MAT”) and ABS agreements (United Nations, 1992: Art 1). From the CBD’s inception, IPLC rights and interests deriving from the use of their own genetic resources and TK were excluded from its scope. This omission was addressed later in 2010 by the supplementation to the CBD of the Nagoya Protocol (“NP”), which entered into force in October 2014. This framework specifically commits Nation States to “respect, preserve and maintain knowledge, innovations and practices” of IPLC, and to uphold ABS derived from their use [Sherman and Henry, 2020; Bavikatte and Robinson, 2011; United Nations, 1992: Art 8(j)]. A fundamental premise of the NP is that IPLC must be viewed as partners rather than sources of biological research (Garrison et al., 2019b). As a result, ABS and the sustainable use of IPLC genetic resources are inextricably linked as one of the CBD’s central objectives (Rodrigo et al., 2022).

2.2 However, the NP as it currently stands protects only physical samples and associated TK, and it remains unclear whether and how digital data, such as DSI should be governed (Ambler et al., 2021; ABS Capacity Development Initiative, 2021b). Consequently, it was not included as part of the NP negotiations due to perceived legal and technical difficulties (Schroeder et al., 2018). Today, however, DSI is essential to innovation and discovery in the life sciences, and it follows that the key principles of ABS, as enshrined by the NP, and consistent with the CARE Principles for Indigenous Data Governance, must be extended to DSI of biological samples if the NP is to continue to carry any weight (Carroll et al., 2020; Carroll et al., 2021).

2.3 Current negotiations concerning the inclusion of DSI entails defining its scope, and clarifying whether it is possible to track DSI within an open data environment (United Nations Convention on Biological Diversity Ad Hoc Technical Expert Group on Digital Sequence Information on Genetic Resources, 2020; Rodrigo et al., 2022). Open data and open science have become commonplace, with many funding sources and scientific journals expecting biological data to be publicly accessible at the time of publication (McCartney et al., 2022). Subsequent use of these data is often unrestricted and unbound by agreements or consent arrangements with IPLC (Garrison et al., 2019b; Ambler et al., 2021). In light of the growing scale of digitization projects, data re-use, and sharing, the limitations of data infrastructures to record Indigenous provenance emerges as a key barrier in establishing the conditions for benefit sharing and supporting IPLC self-determination (Woods, 2018). In view of the ease with which DSI, like all other digital material, is shared across borders, how it is, and should be, considered under the NP’s ABS principles remains obscure (Tsioumani, 2018; Heinrich et al., 2020).

## Issues for Indigenous peoples and local communities within open data frameworks

3.1 Open access to data and associated metadata challenges values expressed through the Indigenous data sovereignty (“IDSov”) movement, which advocates for greater Indigenous control of IPLC data (Kukutai and Taylor, 2016; Davies et al., 2019). The IDSov movement aims to protect the collective interests of IPLC by ensuring the rights of “data for governance” and “governance of data.” It anticipates active Indigenous participation in governance, and serves as an opportunity for IPLC to practice their own norms and values (Geary et al., 2013; Kukutai and Taylor, 2016; Rainie et al., 2017; Te Mana Raraunga, 2018; Walter et al., 2018; Garrison et al., 2019b; Davies et al., 2019; Tsosie, 2019). The principles of free, prior and informed consent, MAT and ABS are key to the NP. However, these concepts are also generally applicable to many different types of Indigenous data and especially relevant to IDSov (Kukutai and Taylor, 2016; Mc Cartney et al., 2022).

3.2 Open data is important to facilitate discovery and innovation, however the resulting impediments to ABS also need to be addressed (Scholz et al., 2020; Lyal and Zhao, 2021; Sherkow et al., 2022). Unrestricted, open access to data effectively removes the need for ongoing consultation with IPLC and, therefore, removes the opportunity to mitigate harms, discuss benefits, or address issues of equity and autonomy (Hudson et al., 2020a). Thus, a more flexible “open as possible, closed as necessary” position towards open data is more consistent with the aspirations of IPLC to govern the use of data encompassing their TK and resources (Rainie et al., 2017; Te Mana Raraunga, 2018).

3.3 Misappropriation of DSI and other digital TK is analogous to unethical bioprospecting, which remains a pervasive issue for IPLC genetic resources, especially where reattribution is an arduous and resource intensive process, e.g., in South African Rooibos, Peruvian Camu Camu and Hawaiian Taro (Robinson et al., 2010; Oguamanam, 2018). Extractive research is pervasive in the sciences and leads to mistrust and an unwillingness to enter into partnerships. As a result, the relationship between researchers and communities can be irrevocably damaged, hindering partnerships between researchers and IPLC, risking foreclosed access to study sites, subjects and materials (Ambler et al., 2021; Robinson, 2021).

3.4 Debate over data governance in light of ABS continues, but as a crucial first step, the inclusion of obligations around the disclosure of data provenance could be undertaken as a data quality checkpoint with wide reaching benefits, independently of governmental leadership (Buckner et al., 2021; McCartney et al., 2022). This can take place at multiple points of the data cycle. For instance, researchers and administrators of data repositories can play an immediate role by ensuring genetic data are attributed to IPLC by appending metadata to database submissions using already available solutions, such as the now widely-used TK Labels (Anderson and Christen, 2019; Montenegro, 2019; Hudson et al., 2020b).

## The Nagoya Protocol and Indigenous data sovereignty need not impede research

4.1 A common argument made by supporters of open science and open access is that the inclusion of DSI in ABS protocols will “stifle research and innovation” (Rodrigo et al., 2022). Proponents assert that enabling inclusive decision-making will create bureaucratic barriers that would negatively impact the IPLC communities and ecosystems that many researchers strive to protect (Karger and Scholz, 2021). Others argue that furthering collaboration and the sharing of resources facilitates scientific discovery and increases research impact (McKiernan et al., 2016; Allen and Mehler, 2019).

4.2 IDSov does not aim to impede scientific activities, but rather to ensure appropriate Indigenous participation in research concerning their TK, lands and resources (Garrison et al., 2019a). This right is articulated by the United Nations Declaration on the Rights of Indigenous Peoples (2007) and has been affirmed by the Conference of the Parties (“COP”): Decision 14/16 emphasized the importance of “the holistic collective actions” of IPLC, and invited COP members to recognize and fully include traditional knowledge as complementary to Western conceptions of knowledge (United Nations Convention on Biological Diversity Conference of the Parties, 2018). Notably, neither COP reports nor IDSov advocates demand a halt to research, or use of data, concerning IPLC subjects (United Nations Convention on Biological Diversity Open Ended Working Group, 2022). Instead, they invite IPLC members and researchers to engage in open dialogue on issues including MAT and ABS as a crucial step toward the recognition of IPLC self-determination and autonomy. The lack of inclusion of DSI within the NP concurrently has the potential to undermine the spirit and principles of the Protocol itself, as well as the fair sharing of benefits in general (Karger et al., 2019).

## Digital sequence information benefit sharing policy options

5.1 Current international negotiations at the CBD about including DSI within the scope of the NP’s provisions for fair and equitable ABS of genetic resources have identified six key elements:

- regulating access;
- applicability of prior informed consent;
- requirement for MAT;
- linking benefit sharing to DSI;
- requirement for tracing of country of origin; and
- acceptance of bilateral or multilateral approach to benefit sharing.

**FIGURE 1:**
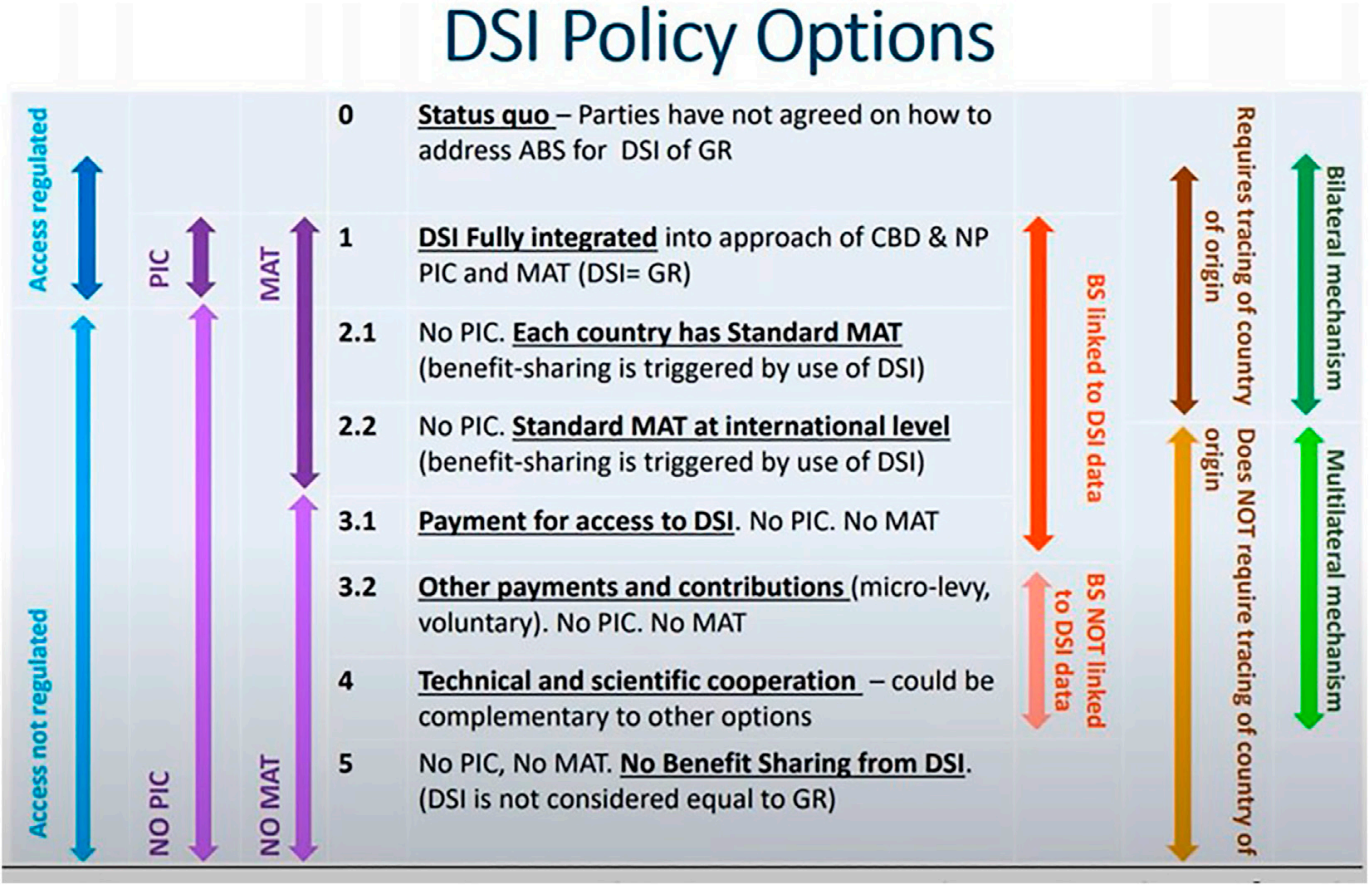
DSI policy options (United Nations Convention on Biological Diversity Open Ended Working Group, 2021).

5.2 These elements have created a set of policy options for DSI reflected in Figure 1.

5.4 The primary benefit sharing approaches currently being discussed are either: a bilateral mechanism wherein benefits are distributed directly to Nation States by tracking and tracing DSI provenance; or a multilateral mechanism where benefits are distributed *via* a general pool, which could be operationalized in the absence of provenance metadata. The multilateral mechanism does not regulate access to data, require tracing of country of origin, or negotiate MAT by country (United Nations Convention on Biological Diversity Open Ended Working Group, 2021).

5.5 Most DSI data records on open data repositories currently do not list country of origin, let alone information about Indigenous communities (Scholz et al., 2021). This failure to include proper provenance or attribution to IPLC is a larger problem for research, and pervades many archives, museums and specimen collections that now must confront the values of past researchers and institutions (Anderson, 2012; Anderson and Christen, 2019). This lack of disclosure creates limitations to accountability and the possibility of future relationships. In the context of DSI, it reduces researcher’s accountability in the future use of the DSI data, and removes the opportunity for countries, or IPLC to benefit directly from the use of genetic data generated from their lands and territories.

5.6 The importance of provenance metadata is recognized as useful for both scientific and equity outcomes by operationalizing bilateral ABS while also enabling innovation and discovery (Buckner et al., 2021; Liggins et al., 2021; Scholz et al., 2022). While Indigenous provenance metadata supports ABS, its primary function is to provide future opportunities for researchers and communities to engage directly and to build relationships and capacity (McCartney et al., 2022).

## Recognizing Indigenous provenance in metadata using biocultural and Traditional Knowledge Labels and notices

6.1 The exclusionary nature of intellectual property limits its usefulness as the mechanism for recognizing cultural authority and Indigenous rights (Anderson and Hudson, 2020). Similarly, IPLC are not in a position to use mechanisms such as Creative Commons licenses; such tools depend on the community first holding copyright in the DSI. However, the Traditional Knowledge Labels (“TK Labels”) and associated mechanism of Notices are an international digital labeling system that records provenance information within metadata. As extra-legal instruments, the Labels and Notices make it possible for researchers to disclose Indigenous interests, and for IPLC to affirm the nature of their relationship to the data as well as protocols and permissions for re-use. The Labels and Notices do not require legislation to be operationalized, making them accessible and immediately available to address IPLC cultural and property interests. The Labels and Notices are part of the Local Contexts system which ensures relevant information about IPLC remains associated with data as it is shared (Anderson, 2012; Anderson and Hudson, 2020; Liggins et al., 2021; Mc Cartney et al., 2021).

6.2 TK Labels were developed to ensure cultural authority could be represented on digital records pertaining to traditional knowledge and cultural heritage items. The 20 TK Labels evolved over the past 10 years in partnership with international Indigenous communities, reflecting community expectations in relation to provenance, protocols and permissions. While the Labels have standardized icons and are intended to ensure international interoperability, the specific Label text is customizable by each community in line with already existing community rules, governance and protocols for using, sharing and circulating knowledge and data (Local Contexts, 2022). The TK Labels are being used in a number of institutions, including the Library of Congress in the US. The Labels have permanent identifiers, are machine readable, and can be accessed *via* an open API (see also https://github.com/jacobgolan/enRich).

6.3 While TK is the trigger for Indigenous rights to fair and equitable benefit sharing in the NP, TK is not held within genomic databases like NCBI. Similarly, Indigenous protocols for TK are specific to TK and are not necessarily appropriate for DSI. The BC Labels were developed to offer a practical option for connecting provenance information with DSI submitted to genomic databases. This allows researchers to disclose Indigenous interests in data and enable IPLC to identify key relationships and provide permissions for future use. The ten BC Labels function at the metadata level, providing transparent notice of ethical use of data and contact information for MAT or benefit sharing should other commercialization possibilities arise.

**FIGURE 2:**
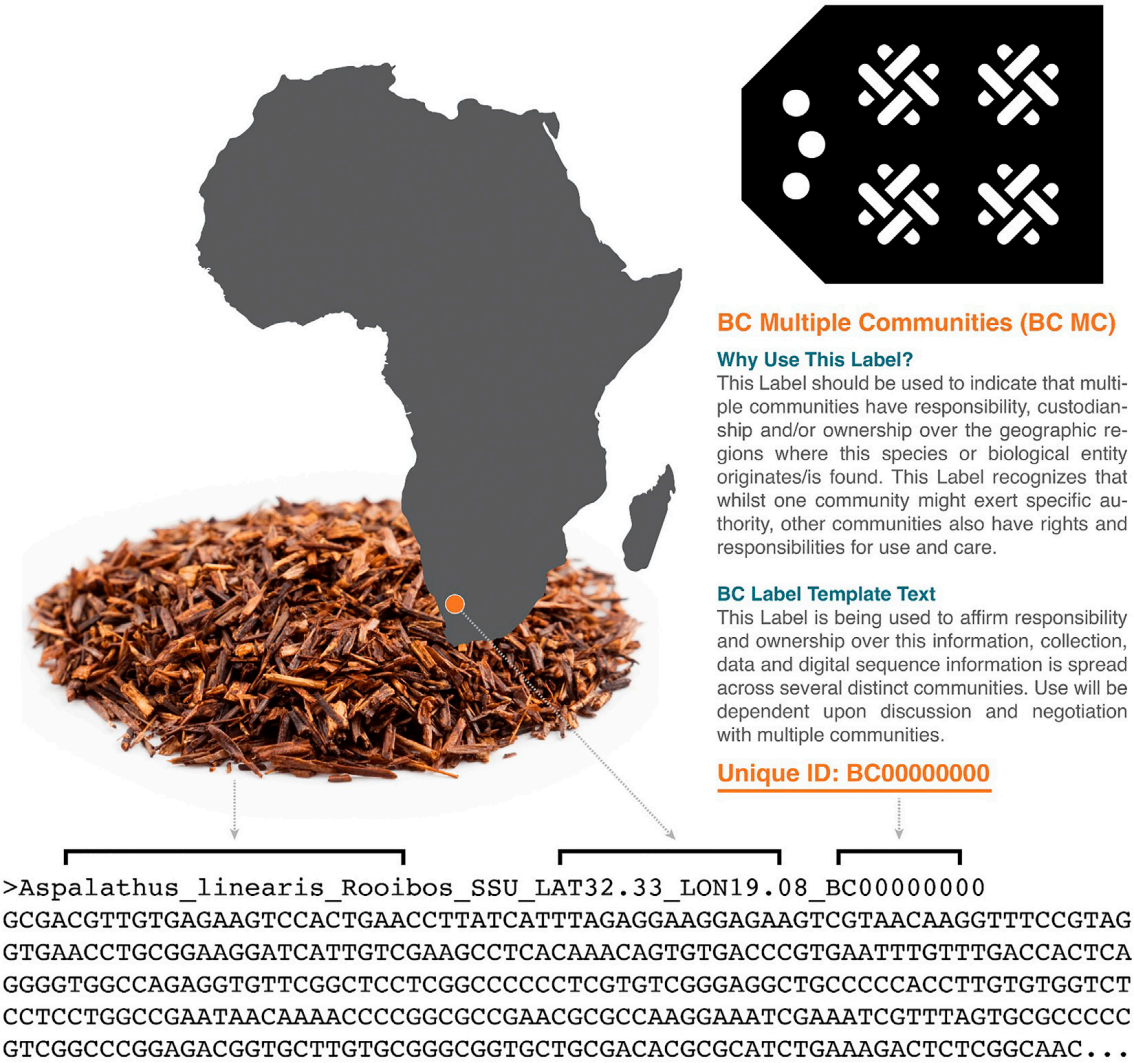
Example FASTA sequence with BC Multiple Communities Label as provenance information included in the header. Provenance information can be appended using the R package enRich (https://github.com/jacobgolan/enRich).

6.4 Metadata is arguably as important as the data itself, as it provides essential context for any information. Like the TK Labels, BC Labels are also designed to be customized by the community so that researchers submitting sequence information to databases can properly attribute and connect provenance to their data (ABS Capacity Development Initiative, 2021a). Additionally, each Label generates a unique ID that can be included in metadata databases or maintained alongside DSI. Appropriate fields for IPLC provenance and/or Labels require that information can be added when uploading raw data to public repositories such as NCBI (National Center for Biotechnology Information), SRA (Sequence Read Archive), GEO (Gene Expression Omnibus) and GDC (Genomic Data Commons). Already however, researchers can append metadata relating to BC Labels and Indigenous provenance to e.g., a FASTA file header (Figure 2).

## What else is needed to understand the value of provenance information to benefit sharing for DSI?

7.1 Despite the central aim of the NP to ensure fair and equitable benefit sharing, discussions at the CBD have focused more on defining the scope of DSI than on exploring how benefit sharing of DSI might be enabled. In order to enhance real-world ABS outcomes for IPLC, it is important to investigate what researchers’ and administrators’ value in access and use of Indigenous DSI. There is a paucity of existing research on ways to measure its market value (Blackwell et al., 2019). Research into the desirability of provenance information assists in the calculation of the market value for genetic resources and TK in existing and future markets (Blackwell et al., 2019). In the case of DSI, the market value is context sensitive, and depends on how fit for purpose the DSI is, as well as the priorities, ethical beliefs and values of the researcher and their organization.

7.2 Modelling the economic value of provenance could be especially useful here, where no market information yet exists for IPLC-attributed genetic resources (Shepherd et al., 2007). Such models must consider the different contexts in which DSI is used, for example simulations of testing scenarios might measure:

- Data integrity, in relation to free and prior informed consent for use;
- Data provenance information to country or IPLC level;
- Data accessibility;
- Future ABS obligations; and
- Amount the user is willing to pay.

7.3 This combination of provenance metadata and valuation models for DSI will ultimately inform the development of regulatory processes for including DSI within ABS provisions in the CBD.

## Conclusion

Including ABS standards for the use of DSI has created a number of practical and technical challenges for policy makers as evidenced by the continuing negotiations at the CBD. Establishing Indigenous data provenance within DSI is central to IDSov. Reclaiming control of data, data ecosystems and data narratives in the context of open data and open sciences is the key focus of the IDSov movement. Framed through control, collective benefit and equity, IPLC need to be repositioned from subjects of data extraction to self-determining creators, users and primary decision-makers. This repositioning must have regard to the production, storage and future use of data affecting Indigenous lives, cultures and environments. If IPLC are not connected to their information and data, then there are limits to how IPLC can govern, make decisions and derive benefits from its future use.

## Data Availability

The original contributions presented in the study are included in https://localcontexts.org/tk-label-hub/ and https://github.com/jacobgolan/enRich, further inquiries can be directed to the corresponding author.
